# Cardiac troponin I elevation with supraventricular tachycardia: two case reports and review of the literature

**DOI:** 10.1186/1756-0500-7-136

**Published:** 2014-03-11

**Authors:** Feng Xue, Ting-Bo Jiang, Bin Jiang, Xu-Jie Cheng, Yong-Ming He, Xun Li, Xiang-Jun Yang

**Affiliations:** 1 Department of Cardiology, First Affiliated Hospital of Soochow University, Suzhou 215006, China

**Keywords:** Cardiac troponin I, Acute coronary syndromes, Paroxysmal supraventricular tachycardia

## Abstract

**Background:**

Although cardiac troponin I gives excellent accuracy in the identification of myocardial necrosis, it can also be elevated in a series of diseases other than acute coronary syndromes.

**Case presentation:**

We present two cases of Chinese patients with a high serum troponin I level after an acute episode of paroxysmal supraventricular tachycardia with normal coronary arteries via angiography.

**Conclusion:**

Abnormal troponin elevations can be seen in patients presenting with paroxysmal supraventricular tachycardia and angiographically-normal coronary arteries. Caution is advised with the use of invasive assessments such as coronary angiography in the differential diagnosis of patients with paroxysmal supraventricular tachycardia and elevated troponin levels.

## Background

Cardiac troponin I (cTnI) is only detectable in adult cardiac tissue [1]. It is a sensitive and specific biomarker consistent with cardiac damage and plays a primordial role in the diagnosis of patients with acute coronary syndromes (ACS) [2]. A high cTnI level however is not synonymous to ACS [3]. Patients with normal coronary arteries may have elevated cTnI because of other diseases such as severe congestive heart failure, myocarditis, cerebrovascular accident, renal insufficiency and so on [4]. Since 2003 it has been reported that patients suffering from paroxysmal supraventricular tachycardia (PSVT) had elevated troponin levels [5]. Coronary angiography is often performed in some patients presenting with PSVT because they have chest pain and high troponin levels. Here, we report two cases of PSVT-induced elevations in cTnI in the absence of coronary artery disease.

## Case presentation

### Case 1

**Figure 1:**
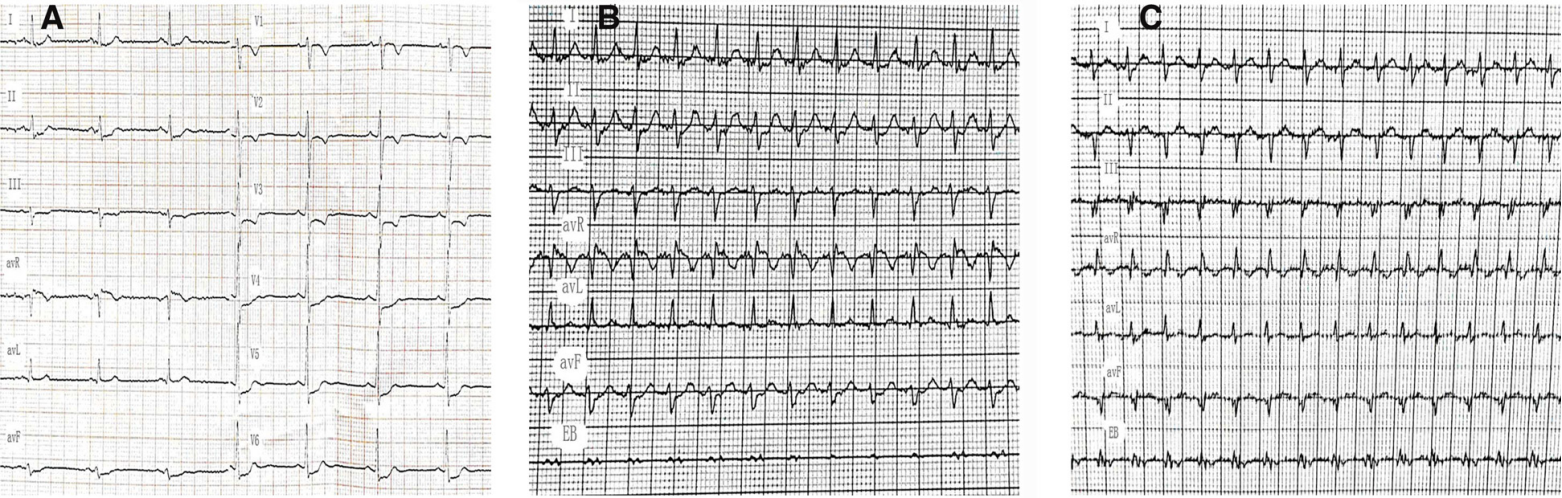
**Electrocardiographic images: The electrocardiogram of case 1 on the admission (A); The result of a transesophageal electrophysiological study of case 1 (B); The result of a transesophageal electrophysiological study of case 2 (C).** (EB: bipolar esophageal lead).

**Figure 2:**
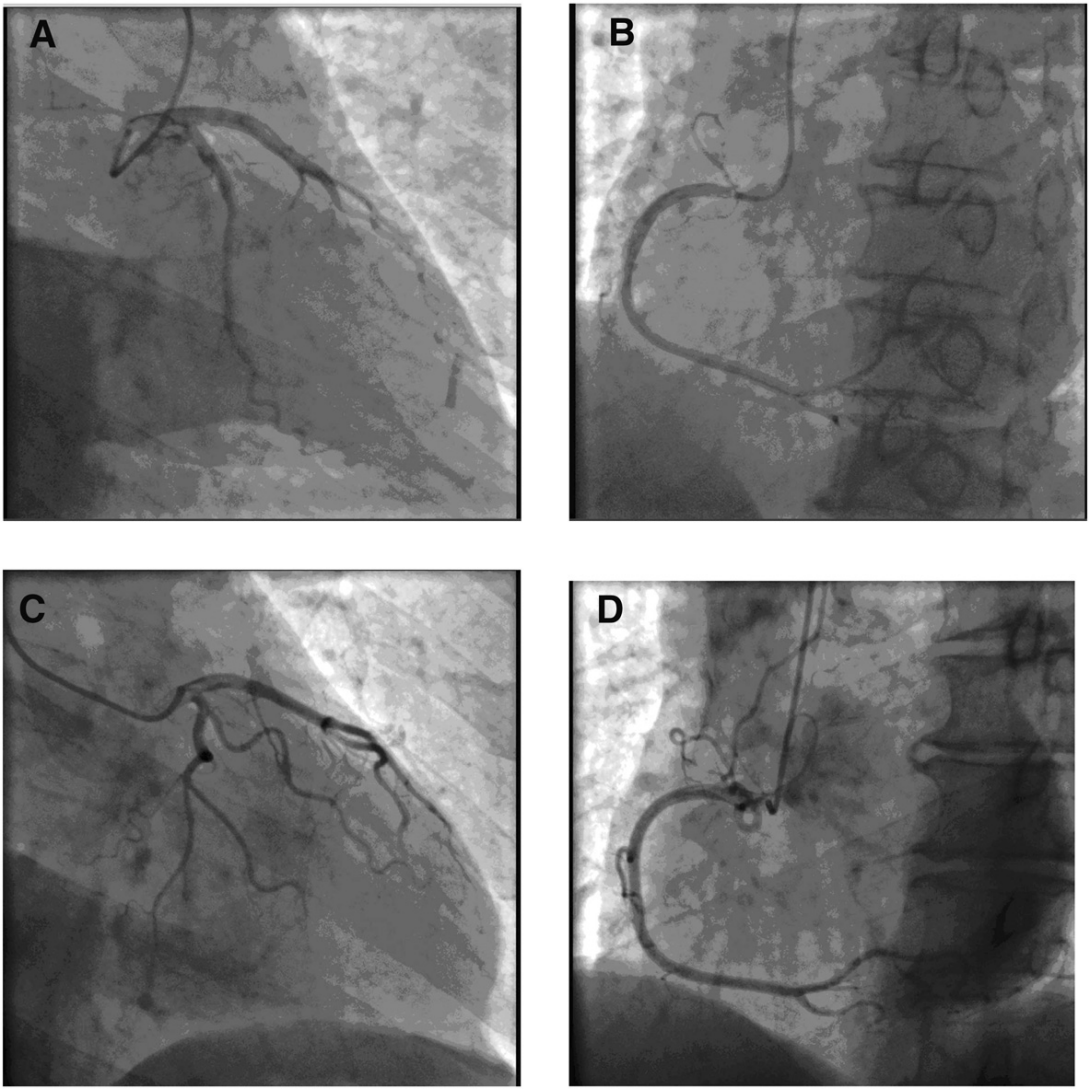
Angiographic images: The left coronary (A) and right coronary (B) of case 1; The left coronary (C) and right coronary (D) of case 2.

A 64-year-old Chinese male patient was admitted to our hospital because of chest pain and palpitation on April 14, 2012. The symptoms lasted for forty-five minutes and were relieved when he arrived at the hospital. He had no history of hypertension, diabetes mellitus or other heart diseases. The results of physical examination were normal. His electrocardiogram showed ST segment depression > 1.0 mm in leads V_3-6_ (Figure 1A). The echocardiogram showed no abnormal changes. The level of cTnI was 0.09 ng/ml (normal < 0.08 ng/ml). The next day this value was up to 0.52 ng/ml. On the third day the value was down to normal. He was diagnosed as ACS and treated with aspirin, clopidogrel, low molecular weight heparin, atorvastatin and so on. One week later the patient underwent a coronary angiography which did not show us any evidence for coronary artery obstruction (Figure 2A,B). During electrocardiogram monitoring transient supraventricular tachycardia was found. Then a transesophageal electrophysiological study was carried on him. A regular supraventricular tachycardia due to reentry within the atrioventricular node was induced and terminated (Figure 1B). At the 15th day of his hospitalization he underwent a successful radiofrequency ablation. Thus his diagnosis was revised as paroxysmal supraventricular tachycardia and he was discharged with no further treatments. During one-year follow-up he was well.

### Case 2

A 66-year-old Chinese male patient with recurrent palpitation for more than ten years was hospitalized with chief complaint of chest pain and palpitation for eight hours on Feb 27, 2013. A minimal response to sublingual nitroglycerin was seen. There was no abnormal changes in the electrocardiogram and echocardiogram. The level of cTnI was 0.16 ng/ml on admission. Nine hours later the value was up to 2.28 ng/ml. He was also diagnosed as ACS and received the same medicine as the first case. On the second day after hospitalization he felt palpitation again. The electrocardiogram showed supraventricular tachycardia that was terminated by the transesophageal atrial pacing (Figure 1C). The esophageal electrocardiogram demonstrated that he had atrioventricular nodal reentrant tachycardia. In order to determine if he had coronary artery disease, we performed a coronary angiography on this patient on Mar 1, 2013. Cardiac catheterization demonstrated normal coronary arteries (Figure 2C,D). After undergoing a radiofrequency ablation he was discharged from our hospital. As of the time of this writing he has no sympotoms.

## Discussion

Paroxysmal supraventricular tachycardia is a common and generally benign arrhythmia. It rarely results in any adverse clinical outcomes. But it was reported that 30% of patients presenting with PSVT had significant troponin elevations [6]. Because these patients had symptoms of chest pain and chest discomfort they were often misdiagnosed as ACS and given inappropriate treatments such as antiplatelet and antithrombotic therapy. Coronary angiography however revealed that most of them had normal coronary arteries. The mechanism of tachycardia-induced troponin elevation is not fully understood [6]. Most authors are in agreement with the mechanism that tachycardia increases myocardial oxygen demand, while it decreases myocardial oxygen delivery because of short diastole during which myocardial perfusion occurs. So tachycardia ultimately leads to reduced myocardial perfusion which results in the release of cTnI into the circulation [4].

What are the characteristics of patients with elevated troponins due to PSVT? We reviewed the English-language scientific literature from the MEDLINE and ELSEVIER databases using the keywords “troponin” and“PSVT”. Most authors found that patients with elevated troponins after acute attack of PSVT were not at increased cardiovascular risk such as hypertension, diabetes or dyslipidemia [6]. This agrees with our cases. They also believed that the duration of arrhythmia was not associated with troponin elevation [7]. However, the maximal PSVT heart rate, ST-segment depression ≥1 mm during the episode of PSVT and the presence of impaired left ventricular systolic function were correlated with troponin elevation [6,8].

Is coronary angiography necessary to be carried out in these patients with PSVT which resulted in elevated troponins? Dorenkamp’s study replied this question [9]. In their retrospective analysis troponin levels were increased in 14 of 114 patients. Thirteen of the 14 patients were subjected to coronary angiography. The result was that no one had significant coronary stenosis. They found a positive exercise test was the best predictor of significant coronary artery disease and subsequent revascularization. A history of hypertension and age 60 years or more ranked secondly. So when we confronted a patient with PSVT and elevated troponin, we should pay attention to his medical history including cardiac risk factors and age. Then a noninvasive assessment for coronary stenosis such as the exercise test can be chosen before coronary angiography. This was not implemented well in clinical practice according to our cases and other reports.

What is prognostic significance of elevated troponins in these patients with PSVT? This question has different answers. Calberg and his colleagues made a retrospective review of 51 patients with PSVT, 38 of whom had cTnI value meassured at least one time [4]. Eleven patients having elevated cTnI were followed up for thirty days. They found that none of patients had adverse cardiovascular outcomes. On the contrary, Chow and his colleagues’ research drew the conclusion that mild cTnI elevation in patients with PSVT was associated with increased risk of future cardiovascular events such as death, myocardial infarction or cardiovascular rehospitalization [8]. They studied 78 patients including 29 patients with elevated cTnI. The mean follow-up period was 2.2 ± 1.7 years. These two retrospective studies had a common limitation that the numbers of patients were too small for analysis. Both prospective observational studies and larger retrospective studies are recommended to answer this question.

## Conclusions

Abnormal troponin elevations can be seen in patients presenting with PSVT and angiographically normal coronary arteries. Patients can not be diagnosed as ACS only because they have elevated troponins. Caution is advised with the use of invasive assessments such as coronary angiography in the differential diagnosis of patients with PSVT and elevated troponins.

## Consent

Written informed consent was obtained from both patients for publication of this case report and any accompanying images. A copy of the written consent is available for review by the Editor-in-Chief of this journal.

## Abbreviations

CTnI: Cardiac troponin I; ACS: Acute coronary syndromes; PSVT: Paroxysmal supraventricular tachycardia.

## Competing interests

The authors declare that they have no competing interests.

## Authors’ contributions

FX analyzed and interpreted the patients’ data and was a major contributor in writing the manuscript. TBJ and BJ performed the radiofrequency ablation on the patients. XJC and YMH performed the coronary angiography on the patients. XL and XJY evaluated the draft and suggested revisions. All authors read and approved the final manuscript.

## Authors’ information

All authors are staff members of the Department of Cardiology, First Affiliated Hospital of Soochow University, Suzhou, 215006, China.
